# The Estimand Framework in Diagnostic Accuracy Studies

**DOI:** 10.1002/sim.70248

**Published:** 2025-09-17

**Authors:** Alexander Fierenz, Mouna Akacha, Norbert Benda, Mahnaz Badpa, Patrick M M Bossuyt, Nandini Dendukuri, Britta Rackow, Antonia Zapf

**Affiliations:** ^1^ Institute of Medical Biometry and Epidemiology University Medical Center Hamburg‐Eppendorf Hamburg Germany; ^2^ Clinical Development and Analytics Novartis Pharma AG Basel Switzerland; ^3^ Department of Medical Statistics University Medical Center Göttingen Göttingen Germany; ^4^ Department of Epidemiology and Data Science Amsterdam University Medical Center, University of Amsterdam Amsterdam the Netherlands; ^5^ Department of Epidemiology and Biostatistics McGill University Montréal Quebec Canada; ^6^ Institute of Health Economics and Health Care Research University Medical Center Hamburg‐Eppendorf Hamburg Germany

**Keywords:** diagnostic accuracy study, estimand, ICH E9 addendum, index test, interfering event, missing values

## Abstract

Diagnostic accuracy studies evaluate how well a diagnostic test can detect or rule out a medical condition. Different events can interfere with the conduct of the test, affecting the test result. Before starting a diagnostic test accuracy study, the clinical question of interest should be precisely defined. Based on that, strategies can be chosen for dealing with the interfering event. We introduce six different strategies for how such events could be handled. We introduce the estimand concept for diagnostic accuracy studies, which consists of the attributes population, target condition, index test, accuracy measure, and the strategies for handling interfering events. The estimand determines which effect is estimated based on the study objective. To bridge the gap between the clinical study objective and the method for the estimation, we illustrate the necessary steps using a fictitious computed tomography scan study. The defined estimand improves the structure of the planning phase, enhances the interdisciplinary exchange, and supports the interpretation based on the study objective.

## 1 Introduction

Diagnostic tests use different types of patient information, such as symptoms, laboratory values, or physical examinations, to assess the health status of a patient [1]. Diagnostic accuracy studies are carried out to evaluate how accurately a diagnostic test procedure detects or rules out a medical condition (in the following referred to as disease status) in real life. During the planning phase of a diagnostic test accuracy study, it is essential to clearly define the specific test to be evaluated, including the possible outcomes (e.g., test decisions and procedure to derive test decisions), the target condition, and the population being studied. The precise definitions help to formulate the clinical question of interest and characterize the objective of the trial. However, if, for example, patients with a more severe stage of the disease are selected for study inclusion and hard‐to‐diagnose patients are excluded, there is a risk of falsely overestimating the accuracy of the test [2]. This selective inclusion may result in higher test accuracy compared to its real‐world application. Therefore, it is of great importance to clearly define the latter application and to reflect this in the study when estimating diagnostic accuracy.

To address the precise target of estimation in therapeutic studies, the International Council for Harmonization of Technical Requirements for Pharmaceuticals for Human Use (ICH) introduced the concept of estimands in the addendum to guideline E9 in 2019 [3]. They define the estimand as the treatment effect of interest based on the clinical trial objective, consisting of five key attributes: Treatments, population, endpoint, population‐level summary, and strategies for intercurrent events (ICEs). An ICE is defined as an event that occurs after the start of treatment and influences the measurement interpretation or the existence of values. The guideline suggests five disparate strategies to handle ICEs, each addressing a different clinical objective. The estimand concept for therapeutic studies is an ongoing research field with various methods for handling ICEs and implementations to different study designs [4].

In a diagnostic accuracy study, the test result determines whether a patient is classified as diseased (positive) or healthy (negative). However, various events during the study can influence this classification process. Some factors modify the test procedure, such as additional information, while others may prevent obtaining a test result, like early termination. Such incidents can be informative or non‐informative. For example, in the context of interpreting a CT scan in subjects suspected of lung carcinoma, early termination due to arrhythmic breathing would be informative. Early termination due to claustrophobia would be non‐informative in this context. These deviations must be accounted for appropriately, as they can introduce bias and compromise the validity of accuracy estimates. We denote those occurrences as interfering events (IE). To handle these IEs, the test decision can be, for example, taken over, defined as positive/negative/inconclusive test results, predicted, or completely ignored. The interplay between the clinical question of interest and those events should be considered. In the definition of both the test decision and the measure describing the properties of the test, each event can be handled differently, and the mix of those forms a crucial element of the trial objective.

As mentioned, IEs can lead to an affected test result or a non‐existent test decision. In both cases, the chosen strategies determine how to predict the “true decision”. However, missing values can also occur without the occurrence of an IE, for example, if the biopsy sample is lost or the image is accidentally deleted after the test result has been collected. We define missing test results due to an IE as non‐existent results, meaning the test decision is unavailable because of the event. In addition, it is specified how such results are evaluated in terms of accuracy. For instance, if a CT scan image is unavailable because of patient termination caused by coughing, then this result does not exist, but it could be considered as a positive test result. In contrast, if no IE occurred and the image was unintentionally deleted after collection, the result existed but is now considered missing.

IEs can arise throughout the study process. For the index test, researchers should consider how to handle affected and non‐existent test results in clinical practice. This approach should be in line with the strategy chosen for the event. In most diagnostic trials, it is common practice to exclude patients with inconclusive or missing test results from the analysis [5]. This restriction could introduce bias and compromise the accuracy assessment of the evaluated test. Excluding patients with an inconclusive test result, for example, may lead to a restriction of the study population (spectrum bias), which leads to excluding patients with mild disease and patients with severe non‐disease and, accordingly, to an overestimation of sensitivity and specificity [6]. Excluding those patients may result in a deficient external validity of the study results, and the clinical question may remain inadequately answered. Similar issues may arise with the reference standard, where missing or imperfect results are common due to unforeseen challenges. Therefore, the characteristics of IEs must be carefully considered to accurately determine the true disease state.

This article aims to extend the estimand framework to diagnostic trials. The following section explains diagnostic accuracy studies in more detail. In Section 3, IEs and various strategies to handle them are presented and discussed. Section 4 introduces the complete estimand framework for diagnostic studies and uses a motivating example study to define the estimand. Section 5 outlines the process from defining a study objective to getting an appropriate estimate. The differences in the framework for defining a diagnostic comparative study are explained in Section 7. In the last section, the advantages and limitations of the estimand framework in diagnostic studies are discussed.

## 2 General Characteristics of Diagnostic Accuracy Studies

There exist different approaches to classify diagnostic trials. One method is to sort studies by the level of evidence [7]. Another option is to sort them according to the types of questions addressed in this study [8]. In the context of clinical settings, studies are segmented into construction studies, accuracy studies, change in management studies, and outcome studies [9]. Diagnostic test accuracy studies lie between the phase of the technical evaluation of the diagnostic test and the evaluation of the patient‐relevant outcome. In these studies, the accuracy of a diagnostic method is of paramount interest.

A diagnostic accuracy study requires at least two different tests: The new test under assessment, designated as the index test, and its counterpart, the gold standard. It is assumed that the gold standard or reference standard, as an alternative, accurately detects the true disease state of the patient. This allows categorizing the population into healthy subjects (those without the target condition) and diseased subjects (those with the target condition). Test accuracy for the test under evaluation can be calculated in each subgroup. This leads to “sensitivity”, the probability that a diseased patient is correctly identified, and “specificity”, the probability that a healthy patient is correctly identified. Diagnostic accuracy studies can be categorized into single‐test accuracy studies and comparative accuracy studies. In the single‐test accuracy study, the agreement between the index test and the reference standard is evaluated [10], while in a comparative study, the accuracy of two or more tests is compared, based on the reference standard. Different designs exist to compare two or more tests in a study. In the paired design, all tests are examined for each study participant. In the unpaired design, each participant is examined by one of the tests plus the reference standard, ideally randomly assigned [11]. Patient enrolment can distinguish between the cross‐sectional sampling, where the true condition is not known in advance and patients are included based on a set of inclusion criteria [6], and the case‐control design, where the true condition of a patient is already known in advance [2]. In the case‐control sampling, diseased patients are sampled separately from healthy patients [6]. The estimand concept applies to all kinds of diagnostic accuracy studies. The estimand framework will be exemplified for single‐test studies. Extensions to the comparative design are explained in Section 6.

### 2.1 Differences Between Therapeutic and Diagnostic Studies That Call for a Specific Estimand Framework for Diagnostic Tests

Diagnostic studies are fundamentally different from therapeutic studies.

Whereas the outcome of a therapy to be evaluated relates to the health status of a patient, the outcome of a diagnostic test is a decision on the presence or absence of a disease (status). A clinical study to assess a therapy intends to measure an underlying treatment effect. A diagnostic study evaluates the operational characteristics of a decision procedure. ICEs in therapeutic studies refer to events that occur after treatment initiation with a potential influence on the patient‐related outcome, even if not observed in the clinical trial. The target of estimation can be described as the effect of the new treatment concerning the underlying resulting health status of the patient. The estimand strategy decides which true underlying effect is estimated, depending on the ICE.

In contrast to this, the outcome of a diagnostic test relates to a decision after performing the index test, and the event that may interfere with this decision‐making process has no influence on the health status itself, but rather on the test decision (on the health status) to be taken. The accuracy to be estimated depends on the definition of the decision rule on which the test result is based. Hence, we believe that (1) the term IE is better suited for diagnostic studies and (2) the test as a decision rule may incorporate the IE as a way to optimize the test properties, that is, the definition of the estimand of interest also refers to the precise definition of the test itself.

An interfering event (IE) in diagnostic studies resembles an intercurrent event (ICE) in therapeutic studies, but has notable distinctions. In therapeutic studies, the observation period spans a time interval, whereas in diagnostic studies, it is typically limited to the test conduct phase. Additionally, ICEs in therapeutic studies often depend on the treatment group, with one treatment potentially causing more events than the comparator. In contrast, IEs in diagnostic studies are more closely tied to an individual's true disease state, regardless of sampling design (e.g., cross‐sectional or case‐control). For example, patients with the disease may experience test termination more frequently, leading to different event frequencies between diseased and non‐diseased patients. Therefore, it is essential to distinguish IEs in diagnostic studies from ICEs in therapeutic studies.

### 2.2 Example: Computed Tomography Scan for Detecting Lung Carcinoma

In this notional example study, the aim is to evaluate the accuracy of computed tomography (CT) scanning in detecting lung carcinoma.

The first step is defining the clinical trial objective. In the end, the analysis of the study results should answer this clinical question of interest properly. This study specifically investigates the accuracy of the CT scan in suspected cases of lung cancer. Accuracy is evaluated by reliably detecting an existing carcinoma. However, during the CT scan, different IEs can arise, such as intentional motion because of pain, unintentional motion based on arrhythmic breathing, or image degradation caused by clothing, which could potentially lead to an inconclusive result. Other issues like claustrophobia, shortness of breath, or coughing might prompt an early termination, resulting in an absent image. For example, arrhythmic breathing, coughing, or motion because of pain could be informative for the disease, whereas termination because of claustrophobia could be seen as non‐informative.

## 3 Interfering Events in Diagnostic Studies

**FIGURE 1:**
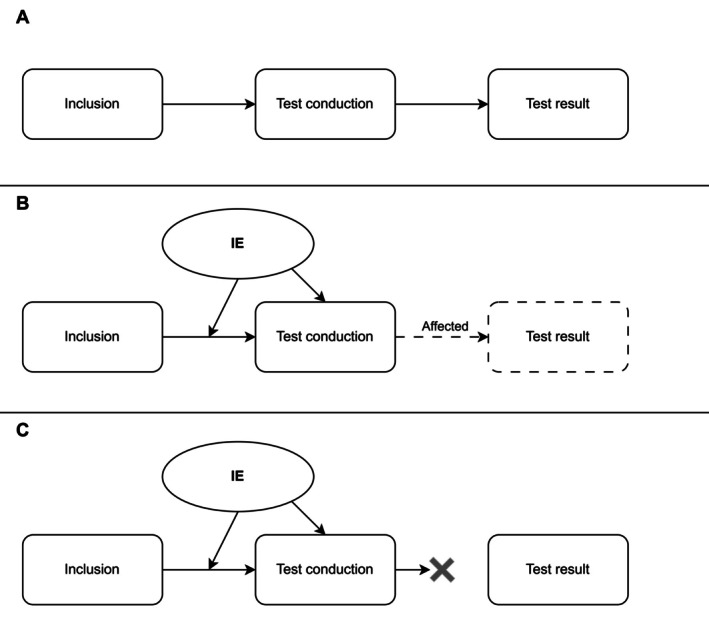
Progression of an individual in a diagnostic study: (A) Without any IE; (B) With an IE that leads to an affected test result; and (C) With an IE that prevents a test result.

An *interfering event* is an incident that occurs after study inclusion, either before or during the test administration, and affects either the test result or leads to a missing test decision associated with the clinical question of interest (see Figure 1).

A disregard of the incidents would change the accuracy measurement. It must be considered to assess the credibility of the test decision in the presence of an incident. If the result is not trustworthy, then the incident must be handled as an IE. This implies that this IE is informative for the test decision and, consequently, for the accuracy assessment.

Various IEs may arise in diagnostic studies. They could be based on occasions on the individual level or on incidents that occur during test administration and interpretation. Some common examples of IEs in diagnostic studies are covered in the following:

- When conducting a diagnostic accuracy study, there is the possibility of an intermediate test result. This means that an individual can neither be classified as diseased nor as healthy. There exists a state between clinically relevant health conditions. It is unknown if this result occurs more frequently in one of the two states. For example, a test may rely on two different parameters, with a positive outcome when both are fulfilled and a negative outcome when both fail. If one parameter is met and another fails, an intermediate test result is observed. Another example is a laboratory value falling between the cut‐offs for a positive or negative test decision, the values are above the measurement range, or below the lower quantification limit. An intermediate result could also be due to an undefined subtype or substate of the disease.
- It is important to distinguish the intermediate from the uninterpretable test result. In the latter, the decision on the health state cannot be made because of the inadequate quality of the test administration. Examples could be the poor quality of an X‐ray image, an insufficient sample amount, or a failed quality assessment of the test administration. In the end, both types of test results lead to an inconclusive test result.
- Another possible IE is accidental unblinding, where the assessing person already knows the other test result, making an objective decision about the evaluation of this unblinded test impossible. If ignored, this results in an overestimation of the accuracy of the index test.
- A further IE is a modified test. For example, in the case of a hard‐to‐diagnose patient, the test is modified by an additional test to verify the test result.
- A treatment started before the test was conducted is also an IE that could influence the test result. In most cases, it is not clear if reliable test results are observed when an individual has started treatment associated with the disease.
- Further common IEs are refusal of the test, which could be linked to the disease state, or interruption/termination of the test. Examples of the latter are patients experiencing panic attacks during an MRI or complications during a blood sample.
- Another IE is a possible comorbidity that could influence the test result. For example, blood samples cannot be assessed due to hemolysis or lipemia.

Beyond those mentioned earlier, additional IEs can manifest, especially test‐specific ones that need to be identified during the planning phase. Therefore, it is crucial to understand the concept of IEs, how to identify them, and which problems could arise if not handled effectively. Possible options for dealing with them are explained in the following section.

### 3.1 Strategies to Handle Interfering Events

We introduce six strategies, inspired by approaches for handling ICEs in therapeutic studies (ICH E9 Addendum) [3], to align IEs with the clinical trial objective in diagnostic studies.

- The *diagnostic policy strategy* accepts the occurrence of events and ignores them during the test procedure. With this strategy, the index test results are used regardless of the occurrence of the IE.
- The *indicative event strategy* considers a possible correlation between the event and the result and is used as an additional option to make the test decision.
- The *setback strategy* assumes that the event adversely influences the test decision, and therefore, the result is assigned to be wrong.
- The *hypothetical strategy* indicates the test accuracy in a hypothetical scenario where the occurrence of this event is not possible.
- The *while under monitoring strategy* is used for index tests with a longer period. Only measurements before the occurrence of the event are employed to predict the result of the index test.
- The *principal stratum strategy* describes the accuracy of the index test in a target population in which a potential IE would or would not occur.

This list of strategies is not exhaustive, and further strategies could be conceivable. The selection of a particular strategy results in a defined clinical question of interest. A change in the strategy alters the estimand. It is recommended to decide on a specific strategy for every single event, but not every strategy is suitable for all IEs. For example, an IE that always leads to a non‐existent test decision (i.e., that the result could not be assigned to any of the categories positive/negative/inconclusive) cannot be handled with the diagnostic policy strategy. The strategies for the IEs must be chosen with caution to ensure that the assumptions align with the clinical question of interest. For the mathematical notation of the strategies, see Appendix A.

One approach to handling an IE is to use the *diagnostic policy strategy*. This strategy accepts that an event could influence the test decision and ignores its occurrence. The index test result is used irrespective of whether the IE occurred. The IE adapts the attribute of the index test and allows the event to occur during the execution, while the index test decision adheres to the predefined decision rule. The advantage lies in evaluating the accuracy of the new test under real‐world conditions. An IE that consistently results in non‐existent test decisions is not suitable for this strategy. If only in some cases, the event results in non‐existent test decisions, then imputation methods under this strategy must be selected. Examples of approaches can be found in treatment studies [12, 13]. This strategy states the diagnostic accuracy of a modified index test through the occurrence of an event.

In the CT scan example, coughing during the procedure is a common challenge that can be managed using the diagnostic policy strategy. In this approach, the test decision is based on the evaluation of a possibly blurred image caused by the unintended motion (coughing). To address this, the CT scan procedure is modified to allow patients to cough during the examination. This enables the test procedure to incorporate the effect of unintentional movement based on coughing, which can lead to false positive or false negative decisions due to image artefacts, into the overall assessment of diagnostic accuracy.

The *indicative event strategy* incorporates the event in the test result and modifies the index test attribute by making the IE integral to the decision rule. This strategy assumes that the event itself provides a hint to the true state of the individual. This information is now added to the available information of the index test. In this strategy, it is determined that the occurrence of an IE always results in a positive or negative test result. This approach can be beneficial if a dependency exists between the disease state and the IE. This strategy states the diagnostic accuracy of a modified index test, added by a further test criterion.

In the CT scan example, a termination due to shortness of breath could be an indicative event. Under this assumption, it is inferred that patients experiencing such a termination are more likely to be diseased. Consequently, these events are interpreted as positive test results. The scan result is then determined either by the rating of the responsible person or by the occurrence of early termination due to shortness of breath, which is consistently classified as a positive result.

The *setback strategy* treats the IE as a negative factor for the index test, meaning its occurrence should be penalized. When an IE occurs, the index test result is set to the opposite of the true disease status, leading to a misclassification. As a result, the index test attribute reflects an (unknown) incorrect result whenever an IE is present. Consequently, the accuracy measure decreases when an IE occurs and deteriorates further as the frequency increases. This means that the accuracy measure attribute considers a penalization of the IEs that occur. Unlike some other strategies, this strategy always provides a test result in case of an IE. This strategy is beneficial when a test should be used, but implementation faces challenges, for example, when individuals of the target population are not allowed to use this test. This strategy states that the diagnostic accuracy of the index test is penalized due to the appearance of the IE.

An interruption of the CT scan due to technical failures could be rated as a faulty performance of the index test. Therefore, the occurrence of such events is classified as a false (either positive or negative) outcome. The efficacy of the index test is subsequently quantified by assessing its accuracy, taking into account the impact of technical problems that occurred during administration.

The diagnostic policy strategy, the indicative event strategy, and the setback strategy can be seen as part of a broader group of composite test strategies. In each of these, the final test result depends only on two components: The result of the index test and whether an IE occurred. This relationship can be described using a function g(I,E), which combines the index test result I and the occurrence of an IE E into a single decision. When the index test result is missing, an imputation method is required under the diagnostic policy strategy to replace the missing value. Similarly, if the reference standard result is missing, imputation is needed under the setback strategy, since the strategy requires knowledge of the true disease state to assign a misclassification.

The *hypothetical strategy* evaluates the accuracy in a scenario without the IE. This means the question is framed within a hypothetical setting where the event cannot occur. The index test attribute is modified to a hypothetical test where the IE cannot occur, and the population attribute defines a population in which the IE does not occur. Patients with IEs that have occurred are not excluded from the population, but are forced not to have this IE. All influenced test results should be identified to build up this scenario. For each of these affected cases, it predicts what the test result would be if the IE did not occur. A non‐existent or affected result could be imputed based on different values, such as baseline values, disease prevalence, or test probability. The index test now comprises a combination of real and hypothetical results. Crucially for this strategy, the hypothetical scenario must be sensible regarding content and clinical relevance. Otherwise, the study would end up in an impractical scenario, assessing accuracy in a way that could never be achieved. This strategy states the diagnostic accuracy of the index test in a hypothetical world where this IE does not exist.

In the context of a CT scan, a claustrophobic seizure during the procedure can be addressed through the hypothetical strategy. In this strategy, the test result for a patient who terminates the scan early due to claustrophobia is hypothetically imputed based on other clinical measurements obtained at baseline. In this case, the estimand provides an assessment of the accuracy of the CT scan in a hypothetical scenario where claustrophobic patients can adhere to the procedure.

The *while under monitoring strategy* exclusively considers measurements observed before the IE. Only unaffected values are utilized to predict the potential test decision, which changes the index test attribute to allow an early evaluation to obtain the test result. It follows that values after the event do not have to be collected, and they are not considered to be missing. This strategy is only suitable for a test with an observation period. If the index test uses repeated measurements or several areas, only the collected observations up to the IE are allowed to make the test decision. This strategy states the diagnostic accuracy of the index test until an IE occurs.

During the CT scan, different segments of the lung are observed. If the patient voluntarily moves during the procedure, the while under monitoring strategy could be used. In this approach, only the images captured before the motion occurred are used to evaluate the health condition of the lung.

In the *principal stratum strategy*, the potential occurring IE is used to define subgroups of interest. The analysis set is therefore based on those potential events, and the population attribute is modified by their occurrences. There exist two possible strata. The first group comprises individuals in whom the IE would not occur. Here, the focus is on assessing the accuracy of the index test when it is not affected by the event. The second group is defined as individuals in whom the IE would occur, focusing on evaluating the accuracy of the index test in all affected individuals. The diagnostic accuracy for this strategy is now evaluated in specific strata.

Whether a diseased person would experience the IE if they were healthy, or vice versa, is not of interest. This is because when a person transitions between health conditions, their diagnostic accuracy is assumed to align with that of other individuals in the same state, with a present or absent IE, depending on the potential IE of the individual. Consequently, the accuracy remains unchanged when considering patients from different states.

A stratum in the CT scan example could be patients who can breathe rhythmically. The IE in this case is arrhythmical breathing. One application of the principal stratum strategy involves modifying the population to include only patients who exhibit rhythmic breathing. The estimand is then exclusively defined within this stratum, which consequently allows for a targeted assessment of the accuracy of the CT scan, specifically within this subgroup. Another approach could involve investigating the accuracy of the test solely within the subgroup of patients experiencing arrhythmical breathing (the IE).

### 3.2 Differences Between Strategies in the CT Scan Example

Some notional numbers for the CT scan study were used to demonstrate the differences between the strategies. The potential IE in the index test is coughing during the CT scan. In this study, 200 patients were recruited, 40 with lung carcinoma and 160 without lung carcinoma. 28 of 40 diseased participants got a positive test result, and 144 of 160 healthy participants tested negative. The test results and the occurrence of the IE are broken down for patients with lung carcinoma in Table 1 and without lung carcinoma in Table 2, respectively. The sensitivity and specificity are calculated for different strategies to handle IEs. It is assumed that the IE deteriorates the accuracy of the test. Hence, more participants with IE can be diagnosed correctly in a hypothetical setting in comparison to the diagnostic policy strategy. Another assumption is that the IE appears randomly. Therefore, the accuracy between participants with and without IE is similar in the hypothetical setting. The while under monitoring strategy assumes lower accuracy for participants with IEs than for those without, as only partial scan data (e.g., a lower number of slices) before coughing remain available. The principal stratum strategy is split up into the strata where the IE does not occur and the strata where the IE does occur. The indicative event strategy is handled with all IE‐influenced cases marked as positive or negative. The results are shown in Table 3.

**TABLE 1:** Test result and occurrence of IE in the 40 participants with lung cancer.

	Without IE	With IE
Positive test result	12	16
Negative test result	4	8

**TABLE 2:** Test result and occurrence of IE in the 160 healthy participants.

	Without IE	With IE
Positive test result	10	6
Negative test result	110	34

**TABLE 3:** Accuracy for the CT scan to detect lung carcinoma when coughing is handled with different strategies: DP…diagnostic policy strategy, HY…hypothetical strategy, WUM…while under monitoring strategy, PS1…principal stratum strategy without IE, PS2…principal stratum strategy with IE, IE+…indicative event strategy with positive rule, IE‐ …indicative event with negative rule, SB…setback strategy.

	Lung carcinoma				No lung carcinoma			
Strategy	Without IE	With IE	Combined	Sensitivity	Without IE	With IE	Combined	Specificity
DP	12/16	16/24	28/40	70%	110/120	34/40	144/160	90%
HY	12/16	18/24	30/40	75%	110/120	37/40	147/160	92.88%
WUM	12/16	12/24	24/40	60%	110/120	28/40	138/160	86.25%
PS1	12/16	‐	12/16	75%	110/120	‐	110/120	91.67%
PS2	‐	16/24	16/24	66.67%	‐	34/40	34/40	85%
IE+	12/16	24/24	36/40	90%	110/120	0/40	110/160	68.75%
IE−	12/16	0/24	12/40	30%	110/120	40/40	150/160	93.75%
SB	12/16	0/24	12/40	30%	110/120	0/40	110/160	68.75%

### 3.3 Handling of Various Interfering Events

In a diagnostic accuracy study, multiple IEs can occur. The estimand covers all important events with a determined mix of strategies for the different IEs. Either a single strategy for all different kinds of IEs or an individual strategy for each event type can be chosen based on the aim of the study. If the aim is to show the performance of the test irrespective of the occurrence of an IE, then all IEs should be handled with the diagnostic policy strategy. In a study to evaluate the test accuracy in patients where the test can be conducted without any issues, a principal stratum strategy would be advisable for all IEs.

If the study question is more complex, it is highly recommended to select an individual strategy for each event type. These strategies should align with the clinical question of interest. It is assumed that an individual can only experience a single IE during the test conduct. The mathematical notation of this generalized form is given in Appendix B.

If more than one IE for an individual is possible, then a solution could be to only take the first event into account. Another option could be to sort the events by priority and choose the event with the highest order [14].

## 4 Estimand in Diagnostic Accuracy Studies

The strategies are selected for each IE to align them to the clinical question, and thus, they are part of the trial objective. Other study parameters, like population and tests, are further components of the trial objective. The estimand framework brings all these elements together to reflect the trial objective.

An *estimand in a diagnostic accuracy study* is a precise description of test accuracy that reflects the clinical question defined by the clinical study objective. In single‐test diagnostic accuracy studies, it summarizes the relationship between the test and the presence or absence of a target condition at the population level. In comparative diagnostic accuracy studies, the estimand specifies the difference in accuracy between the various diagnostic procedures being compared.

The estimand in diagnostic accuracy studies is defined based on the clinical question of interest. It describes the accuracy of the diagnostic test in an abstract setting where all information is known. For example, the knowledge of the true condition of the individuals is constantly accessible in this hypothetical setting. The estimand reflects the general statement derived from the study objective, which is to be obtained with the help of a diagnostic study. However, since this estimand is defined in an ideal scenario, it is impossible to collect all the required data. Therefore, the effective implementation of the study plays an important role and should be aligned with the estimand. It is important to note that the study objective guides the choice of the study design and its conduct. The estimand forms the connection between the study objective and design.

### 4.1 Estimand Attributes

To identify pertinent parts of the estimand framework for diagnostic studies, the STARD (Standards for Reporting Studies of Diagnostic Accuracy) [15] and QUADAS (Quality Assessment of Diagnostic Accuracy Studies) [16] tools were consulted. These were used to identify the important aspects of the clinically relevant questions and to understand possible biases that could arise in diagnostic studies. The estimand consists of five different characteristics, referred to as attributes.

These attributes include:

- The *population* of individuals, denoting the group of people for whom the test is used.
- The *target condition* of a patient, representing the disease state or, in general, the condition of an individual to be detected.
- The *index test* under evaluation, representing the diagnostic procedure to be assessed.
- The *accuracy measure* for the diagnostic test, enabling a combination of values.
- The *strategy for handling interfering events*, specifying the chosen strategy for each IE.

All attributes must be unambiguously described to define the clinical question of interest. The definition of the attributes is interchangeable between different estimands. For the definition of a further estimand, it is possible to change more than one attribute. The attributes are interconnected, meaning that a modification in one attribute could affect the others. For instance, the use of a different index test could lead to a modified population based on the tolerability of the test or other events. Another example is the choice of an accuracy measure. If the attribute accuracy measure is sensitivity, the population is defined as all positive patients. In case the attribute is defined as positive predictive value, only individuals who tested positive in the index test are part of the target population. In other words, a change in at least one attribute results in changes in our estimand and, consequently, in a different clinical question.

Another problem arises if one of the attributes is left out and not specified at the outset of the study. In such cases, a poorly defined estimand is unclear and can lead to uncertainties regarding its estimation because it leads to several possible clinical questions. If an estimand is insufficiently defined, this may lead to a misalignment between the study design and the trial objective. Therefore, it is important to define all the attributes clearly and understandably. This section covers their properties in detail.

One attribute is the *population*. It describes the individuals to whom the test should apply in the future, and for whom it is important to distinguish between different states. Within this part of the estimand, it is crucial to specify the individuals for whom the test is being evaluated. Is there a specific subgroup based on demographic factors like sex, age, or region? Is it intended to include only suspected cases, and if so, how is suspicion defined? This could be based on symptoms, laboratory values, or occupational exposure. The population could also be defined as a specific subgroup for whom screening should be performed. Then, this group also needs specifications, such as the starting age of the recommended screening program. Another consideration is the corresponding setting. The test can be administered a priori or as a follow‐up test. The population can also be tested either in a hospital or in an outpatient setting.

The attribute *target condition* indicates the state of health that the index test under evaluation is aimed to detect. Individuals can be categorized into groups with and without the target condition, within which the accuracy of the new diagnostic test can be assessed. To specify the health condition, it must be posed when the condition is absent and when present. This specification can be described by multiple characteristics. To delineate them, several questions must be answered: Which symptoms are substantial? Are there relevant measurements or laboratory values for this disease? Is there any cut‐off variable to distinguish between the two states? Which subtypes of the disease are meaningful to discover? Is there a progression, and which phases of the disease should be addressed? Is there a particular location that stands out? Finally, when everything is known about a patient, it must be possible to assign the individual to a positive or negative state clearly.

Another attribute of the estimand is the *index test*. In this context, the procedure for conducting the test and the decision rule have to be specified for the index test. It must be clarified how the test is to be used in daily practice and its intended application, whether as a replacement for an existing test, an add‐on, or a triage test [17]. For the description of the conduct, it is important in general to specify which steps should be undertaken and in which order they are executed. It should further be outlined what kind of people will perform these tests in their daily routine, and which instruments are used. The second aspect of this attribute is the rule on which the test decision is based. This emphasizes how the result is generated in clinical practice, whether through a threshold value, a score, a signal on a display, or a subjective judgement based on images, for example. It is also important who is allowed to decide on the result: The person performing the test, the person being tested, or both.

The attribute *accuracy measure* selects the measurement(s) for assessing test accuracy. This could be sensitivity and specificity as a co‐primary endpoint [18]. Both can also be combined in the Youden Index, in the positive or negative likelihood ratio. The prevalence plays an important role if the positive and negative predictive values are of interest. The target population should then be aligned with the population in which the test should be used in practice. When interpreting the probability, the index test and the target condition switch sides, which means the test result now defines both groups. But in the estimand definition, the attributes remain the same because they only describe the rule for the test decision and the definition of the health condition. The change in the evaluation is defined in the accuracy measure attribute itself. For the accuracy measure, area under the curve (AUC), a threshold value is not needed in the index test attribute. Then, it is only relevant which quantity the test decision is based on.

The *strategies for handling the interfering events* specify how influenced test results are managed. We introduced six possible strategies: Diagnostic policy strategy, hypothetical strategy, while under monitoring strategy, principal stratum strategy, indicative event strategy, and setback strategy (see Section 3.1). The first step is to identify possible IEs. These events can occur before or during the conduct of the index test and may influence the result. Such incidents may lead to unreliable results or non‐existent values appearing afterwards. Once all possible events are identified, a strategy for each of them has to be selected to handle these IEs. It is highly recommended to choose an appropriate strategy for every possible IE, which means aligning the strategy with the clinical trial objective. The application of a single strategy for all mentioned IEs should be avoided, but can be sensible in certain settings. It has to be noted that a change in the strategy would change the stated estimand. Therefore, it is necessary to select a strategy aligned with the clinical question of interest. An IE affects accuracy at the theoretical, unobserved test level.

### 4.2 Estimand for the CT Scan Example

An estimand for the CT scan trial is defined to illustrate the estimand framework. The first step is to define the clinical question of interest, as it forms the basis for specifying the estimand. In this case, the study aims to answer the question: “How accurately does a CT scan detect lung carcinoma?”. Based on this question, the researchers spell out this aim in more detail by considering the different attributes. In this step, the IEs have to be taken into account. It must be discussed which IEs can occur during the study and how they should be handled. This must be aligned with the posed clinical question. The estimand could now be defined based on this precise research question. For this example, the estimand is then expressed in words as follows: The study aims to evaluate the accuracy (sensitivity and specificity) of a CT scan in detecting the presence of lung carcinoma among suspected patients aged 30 and above who can breathe rhythmically. Positive test results include instances where shortness of breath and coughing lead to an early termination. Accuracy should be measured in a hypothetical setting in which claustrophobic patients are not a problem for the scan.

The attributes of the estimand can be shaped by the clinical question. The target condition is to identify patients suffering from lung carcinoma, defined by the presence of at least one malignant lesion in the lung, restricted to the lung area, and no symptoms must be present. This condition encompasses various subtypes, including non‐small‐cell lung cancer, adenocarcinomas, squamous‐cell carcinomas, and large‐cell carcinomas. The index test is a thorax and abdomen CT scan. The study participants are given a contrast agent for this purpose. After that, the scan is performed with a computer tomography scanner. A radiologist evaluates the images of this scan and decides if a tumour is present. The objective is to use the CT scan as an add‐on diagnostic tool. The target population includes individuals suspected of having lung cancer, aged at least 30, who have at least two of the following symptoms within the last week before the test was conducted: Coughing, chest pain, hemoptysis, breathlessness, fatigue, anorexia, and weight loss. Moreover, one of the following criteria should be fulfilled: Heavy smoker (at least three pack‐years), exposure to hazardous substances (e.g., asbestos and arsenic), or a history of lung infection (e.g., tuberculosis). The accuracy measure for evaluating the CT scan involves sensitivity and specificity as co‐primary endpoints.

Arrhythmical breathing, early termination based on shortness of breath or coughing, and claustrophobic seizure are identified as IEs for this example. Based on the clinical question of interest, we would suggest the following choice of strategies:

- arrhythmical breathing: Principal stratum strategy
- early termination because of shortness of breath or coughing: Indicative event strategy
- claustrophobic seizure: Hypothetical strategy

For the IE of arrhythmical breathing, a principal stratum strategy is employed, exclusively including patients without this IE. IEs resulting in early termination are handled using the indicative event strategy, where a patient is classified as positive if this event occurs. Individuals with claustrophobia are managed with the hypothetical strategy. The test result is predicted based on demographic and clinical values. A short version is shown in Table 4

**TABLE 4:** Short version of the estimand for the CT scan example.

Attribute	Theoretical estimand
Population	Suspected cases over 30 years of age
Target condition	Lung carcinoma
Index test	CT scan evaluated by a radiologist
Accuracy measure	Sensitivity and Specificity
Strategies for IEs	arrhythmical breathing (principal stratum strategy)
	early termination due to shortness of breath
	or coughing (indicative event strategy)
	Claustrophobic seizure (hypothetical strategy)

After translating the trial objective into an estimand, this has to be applied in a clinical study setting, which may introduce new obstacles. The truth about the medical condition is not known. A suitable test, such as a biopsy, should be selected to determine the true disease state. A biopsy sometimes produces inconclusive test results, so that the condition of an individual cannot be classified. In such cases, a choice must be made on how to evaluate those cases. Furthermore, the study population may differ from the target population due to various restrictions. In this example, the population should be restricted to patients without a severe respiratory compromise or pulmonary artery hypertension, because they have a contraindication for a lung biopsy [19].

## 5 From Estimand to Estimation

The pathway from planning to evaluation of a diagnostic study starts with defining the study objective. From this, the clinical question of interest can be derived, which is the basis for the definition of the estimand. This estimand guides the choice of the estimator, that is, the specific method for the estimation of the numerical result (the estimate), which can be calculated after data collection [3].

An estimand is a construct that defines the effect that should be estimated in a perfect setting. The disadvantage is that this cannot be achieved because it is unobservable. In most diagnostic studies, the target condition cannot be determined with absolute certainty. A reference standard is used instead. The objective of the trial, however, is to evaluate the accuracy of the index test in detecting the target disease. As this is not always possible, the study must use an auxiliary measurement and can only answer how well the index test and reference standard agree.

Different obstacles can occur when the estimand is incorporated into the study design. If the attributes cannot be transferred into the study setup, assumptions must be made. Those can be seen as limitations of the study design. In the following part, the challenges of the different attributes will be addressed.

The population attribute and the selected study population can be different. These restrictions of the study population should be mentioned. For instance, when the reference standard restricts the population based on contraindication, or when the test should be evaluated for all adults, but recruitment takes place where only older people attend.

There could be differences from the predefined index test during the study implementation. For example, in the study, the device could be from a specific manufacturer, or a particular group is responsible for test administration or decision making.

The target condition is the biggest challenge. Because the disease state is unknown, a reference standard is applied instead. It is assumed that the reference standard accurately identifies the target condition in all individuals. Therefore, the reference standard must be suitable for the detection of the disease. The more accurately a reference standard can determine the true target condition, the more reliable the accuracy of an index test can be evaluated. The reference standard is merely a tool at the study level, designed to help identify the underlying disease. The use of a reference standard instead of the true health state is always a limitation and should be mentioned. The aim during planning should be to select a reference standard that reflects the target condition as closely as possible. Sometimes, the best option is not feasible due to time, cost, or ethical restrictions. Then the best available option should be discussed and noted as a limitation of the trial. Multiple tests can be used as a reference standard. For example, in a scenario with two reference standards, one is applied following a positive index test result, and the other following a negative result. Caution is advised here due to different evaluations. Further, a composite test consisting of two or more reference standards can be used with a specific rule to obtain a consensus result.

Another deviation from the estimand definition is the time point of assessment of the medical condition. The estimand assumes that the target condition is known at the time the index test was carried out. A limitation is that the reference standard is measured before or after the index test. It is assumed that the true disease state does not change between both tests and that the reference standard reflects the true state of the patient at the time of the index test. Consideration must be given when the disease state may change, and a longer interval between tests is no longer plausible.

In the reference standard, different issues can also arise. These issues are instructional problems in recording the true disease state and could be diverse. There could be missing data issues, such as lost samples, or structural issues, such as a triage test after an indeterminate result. It is crucial to think about a different handling of these diverse issues. These issues should be aligned with the attribute of the target condition. A different handling of them varies the agreement between the index test and the reference standard. This problem is comparable to the choice of a specific statistical method, because the decision should be aligned with the previously defined estimand.

In the CT scan example, those obstacles should be mentioned. The study population is restricted to individuals without a history of lung carcinoma and a contraindication to biopsy, such as severe respiratory compromise or pulmonary artery hypertension. However, the statement regarding the accuracy of the index test should also apply to this excluded group. The reference standard is a biopsy sample, assuming that the histological examination of the sample confirms the presence of malignant cells. Pathologists examine the sample material and decide based on criteria such as protein activity and cell shape. The biopsy sample must be carried out a maximum of one week after the index test. Another limitation is that the index test is evaluated by an experienced radiologist. However, the study result should only be considered valid if all radiologists are permitted to evaluate this index test.

Suitable methods must be further specified to calculate the accuracy of the CT scan. The calculated estimate of the accuracy aligns with the estimand and provides an answer to the clinical question of interest.

## 6 Comparative Diagnostic Studies

In a diagnostic comparative accuracy study, the estimand defines the accuracy difference between the compared tests. Sometimes, the study population can be different between the compared tests, for example, due to tolerability or feasibility. Then, it has to be decided if the population attribute has to be changed or if those cases must be defined as an IE. In comparative accuracy studies, the definition of the target condition is the same for all patients, and therefore, the chosen reference standard must evaluate the true state for all compared tests. In the definition of the index test, all compared tests must be explained in detail; this includes the procedure of the test and the decision rule. Further, in comparative accuracy studies, the accuracy measure must specify how the tests are compared. It must be decided whether, for example, the difference, ratio, or odds of diagnostic accuracy is important for our clinical perspective.

In a study with different diagnostic tests, the characteristics of the IEs can differ. Some IEs only occur in one of these tests or have a different frequency of occurrence. This leads to a diverse influence on the accuracy of the tests. For IEs that were handled with strategies changing the test procedure or decision rule, it could be advisable to choose different strategies for the index and the comparator test(s). For example, after the occurrence of an IE in the index test, the result could be set to positive, but in the comparator test, it makes more sense to ignore the event. Another difference to the single test design arises in the principal stratum strategy. In a paired design, a potential IE can be observed directly in both tests since each participant is undergoing both. In contrast, in an unpaired design, where a participant is randomized to receive either the index or the comparator test, their stratum membership for the unassigned test is unknown and must be predicted, similar to the situation in parallel‐group trials. For example, if a participant is randomized to the comparator test, their potential IE in the index test must be predicted. A special case arises when the same type of IE is handled using the principal stratum strategy for both tests this results in four potential strata: (1) IE occurs in both tests, (2) IE in neither of them, (3) IE only in the index test, and (4) IE only in the comparator test.

## 7 Discussion

Whilst there is a specified estimand for therapeutic studies [3], it does not exist for diagnostic accuracy studies. This article defines an estimand framework for diagnostic accuracy studies. The estimand must be based on the clinical study objective and should be aligned with the clinical question of interest. An estimand must be pre‐specified at the planning phase of a diagnostic trial. The estimand consists of five attributes: Population, target condition, index test, accuracy measure, and strategies to handle IEs. Occurring IEs during a diagnostic procedure can lead to an affected or non‐existent test result. To handle them, six strategies were introduced.

The estimand improves the structure of the planning phase of a diagnostic study. It supports all stakeholders involved across the entire study process during the planning phase, in discussing the trial design, when conducting the trial, and analysis and interpretation of the study results [3]. Further, it fosters the interdisciplinary exchange between clinicians and statisticians by introducing a common linguistic basis. With the help of the estimand, the study results can be interpreted based on the study objective. Therefore, the gained knowledge can be translated from a study to a real‐world setting. The estimand consequently supports making the study results comparable between different trials. By implementing the estimand into the study design, differences between the defined estimand and the study setting can be carved out. Thereby, various risks of bias can be identified and eliminated or reduced. In the attribute strategies for interfering events, the influence of the IE on the accuracy is described. Different methods can be chosen to handle the IEs. The event is thus aligned with the study objective. The framework can also be extended to include other strategies.

The estimand exists only in a theoretical setting and cannot be evaluated in practice. Caution is therefore required in defining the estimand because some of them are meaningless for a clinical setting. For example, the accuracy in the strata of all terminated individuals does not make sense because, for clinical application, we would never get results for this group.

During the execution of the reference standard, different issues can arise that can lead to a missing value and a missing disease state. On the one hand, it could be argued that these obstacles are measurement problems of the reference standard. They should be handled as missing values because the reference standard is not part of the estimand. On the other hand, we believe that considerations on how to handle them beforehand could be valuable. By anticipating potential issues and determining how to deal with them, the reference standard can be defined more precisely and thus better aligned with the target condition.

The framework supports considerations when a missing value should be imputed and when the individual should not be part of the specific estimand analysis. In cases where an IE is handled with a principal stratum strategy, individuals in which this IE occurs should not be imputed if the stratum without the event is of interest. For the accuracy measure sensitivity, a missing index test should not be imputed to healthy individuals. In contrast, if the positive predictive value is of interest, the reference standard should not be imputed to all index‐positive individuals. This plays an important role in the case of partial verification, whereby the reference standard is applied to a small proportion of those who tested negative on the index test.

Another option for the attribute target condition would be to include the reference standard. Then this attribute would be defined as a condition measured with a specific test. We decided to include only the target condition in this attribute because the objective of the study is to analyze the accuracy of the index test to detect the target condition. Comparison between studies is much easier as the results can be matched if the target condition is the same. Otherwise, a different choice of the reference standard would change the estimand and thus prohibit such comparison. Another problem could arise if different reference standards are applied between individuals. Then this attribute would no longer be unique. By defining only the target condition, issues in the reference standard are not considered to be IEs, as they are not part of the estimand. However, similar strategies could be used to align the results of the reference standard with the target condition.

The estimand framework introduces a new work step in the planning phase, which increases the effort between the different stakeholders and requires proper teamwork among the disciplines involved. The estimand makes various studies comparable, but individual studies usually define a different estimand. This means that they cannot be compared on a one‐to‐one basis, but the difference between them can be easily worked out.

In the future, an analytical approach should analyze the impact of the choice of the strategy as well as the influence of various parameters like prevalence, percentage of events, and dependence between IE and state on the estimand. The goal is to compare estimation targets (estimand) across different strategies. For example, an estimand with an IE handled with a setback strategy is expected to have lower accuracy than one using the diagnostic policy strategy. In addition, suitable methods (estimates) for the different strategies will be examined and integrated with approaches for handling missing values.

We recommend using the estimand framework in the planning phase of a diagnostic accuracy study. It supports the alignment between the study objective and estimation. As a result, the study outcome reflects the clinical question of interest and can be interpreted straightforwardly. We hope that this approach improves the pre‐specification of the clinical question of interest and that the framework results in the incorporation of inconclusive or non‐existent test results in the analysis.

## Conflicts of Interest

The authors declare no conflicts of interest.

## Data Availability

The authors have nothing to report.

## Appendix A Mathematical Notations for Strategies for Handling Interfering Events

The accuracy summary of the co‐primary endpoint of sensitivity and specificity is used to describe the strategies mathematically. In the absence of IEs, sensitivity se and specificity sp are defined as

(A1)
$$\begin{array}{ll} se & =P(I=1|S^{+}) \end{array}$$

(A2)
$$\begin{array}{ll} sp & =P(I=0|S^{-}) \end{array}$$

S is the true state of an individual with $S^{+}$ indicating the target condition is present (diseased) and $S^{-}$ meaning the target condition is absent (healthy). I∈{0,1} denotes the result of the index test, taking the value 1 if the index test decision is positive and 0 if the test decision is negative.

### Diagnostic Policy Strategy

The sensitivity and specificity are given as:

(A3)
$$\begin{array}{ll} se_{dp} & =P(I=1\wedge E=0|S^{+})+P(I=1\wedge E=1|S^{+})=P(I=1|S^{+}) \end{array}$$

(A4)
$$\begin{array}{ll} sp_{dp} & =P(I=0\wedge E=0|S^{-})+P(I=0\wedge E=1|S^{-})=P(I=0|S^{-}) \end{array}$$

with E∈{0,1} is the indicator for the IE in the index test. If the event occurred, the value is 1, and 0 otherwise.

### Indicative Event Strategy

The sensitivity and specificity are given as:

(A5)
$$\begin{array}{ll} se_{ie} & =P(I=1\wedge E=0|S^{+})+P({Ĩ}_{2}=1\wedge E=1|S^{+}) \end{array}$$

(A6)
$$\begin{array}{ll} sp_{ie} & =P(I=0\wedge E=0|S^{-})+P({Ĩ}_{2}=0\wedge E=1|S^{-}) \end{array}$$

with

$${Ĩ}_{2}=\left\{\begin{array}{ll} \text{either}\;0\;\text{or}\;1\; & \text{if}\;E=1\\ I\; & \text{else} \end{array}\right.$$

Based on the defined test result when an IE occurs, two different accuracy measures are possible:

(A7)
$$\begin{array}{ll} se_{ie^{+}} & =P(I=1\wedge E=0|S^{+})+P(E=1|S^{+}) \end{array}$$

(A8)
$$\begin{array}{ll} sp_{ie^{+}} & =P(I=0\wedge E=0|S^{-}) \end{array}$$

when the IE is marked as a positive result and

(A9)
$$\begin{array}{ll} se_{ie^{-}} & =P(I=1\wedge E=0|S^{+}) \end{array}$$

(A10)
$$\begin{array}{ll} sp_{ie^{-}} & P(I=0\wedge E=0|S^{-})+P(E=1|S^{-}) \end{array}$$

when the IE is marked as a negative result.

### Setback Strategy

The sensitivity and specificity are given as:

(A11)
$$\begin{array}{ll} se_{sb} & =P(I=1\wedge E=0|S^{+}) \end{array}$$

(A12)
$$\begin{array}{ll} sp_{sb} & =P(I=0\wedge E=0|S^{-}) \end{array}$$

### Hypothetical Strategy

The sensitivity and specificity are given as:

(A13)
$$\begin{array}{ll} se_{hy} & =P(I=1\wedge E=0|S^{+})+P(I_{hy}=1\wedge E=1|S^{+}) \end{array}$$

(A14)
$$\begin{array}{ll} sp_{hy} & =P(I=0\wedge E=0|S^{-})+P(I_{hy}=0\wedge E=1|S^{-}) \end{array}$$

where $I_{hy}$ is the hypothetical test result of the index test if the IE had not occurred.

### While Under Monitoring Strategy

The sensitivity and specificity for this strategy are given as:

(A15)
$$\begin{array}{ll} se_{wm} & =P(I=1\wedge E=0|S^{+})+P({Ĩ}_{1}=1\wedge E=1|S^{+}) \end{array}$$

(A16)
$$\begin{array}{ll} sp_{wm} & =P(I=0\wedge E=0|S^{-})+P({Ĩ}_{1}=1\wedge E=1|S^{-}) \end{array}$$

with

$${Ĩ}_{1}=I(f(M_{1},\ldots ,M_{T-n}))=\left\{\begin{array}{ll} 1\; & \text{if}\;f(M_{1},\ldots ,M_{T-n})>z\\ 0\; & \text{else} \end{array}\right.$$

and $M_{i}$ with i=1,…,T repeated measurements of the index test at time point i. T is the time point of the intended final test decision of the index test, and n is the number of periods influenced by the IE. Then, T−n is the last measurement carried out before the event occurred. The variable z is the cut‐off score for the function f. I is the indicator function that transforms based on the cut‐off f(θ) into 0 or 1. Therefore, if an observation with an IE exceeds this value z, the test result would be 1. This strategy could be interpreted as a special case of the hypothetical strategy where the index test is based on a follow‐up, and the imputation only uses unaffected measurements.

### Principal Stratum Strategy

We denote E(I) as the potential occurrence of the IE when a subject is evaluated with test I. The sensitivity and specificity are given as:

(A17)
$$\begin{array}{ll} se_{ps1} & =P(I=1|S^{+}\wedge E(I)=0) \end{array}$$

(A18)
$$\begin{array}{ll} sp_{ps1} & =P(I=0|S^{-}\wedge E(I)=0) \end{array}$$

for the strata of individuals without the IE and

(A19)
$$\begin{array}{ll} se_{ps2} & =P(I=1|S^{+}\wedge E(I)=1) \end{array}$$

(A20)
$$\begin{array}{ll} sp_{ps2} & =P(I=0|S^{-}\wedge E(I)=1) \end{array}$$

for the strata of individuals with IE.

## Appendix B Mathematical Notation for Various Interfering Events

The formulas for the different strategies can be merged to define the sensitivity and specificity in a generalized form when a mix of strategies is applied:

(B1)
$$\begin{array}{ll} se & =P(I=1\wedge E=0\;|\;S^{+}\wedge E_{ps}(I))\\ & \;+\overset{N}{\underset{i=1}{\sum }}P({Ĩ}_{i}=1\wedge E=i\;|\;S^{+}\wedge E_{ps}) \end{array}$$

(B2)
$$\begin{array}{ll} sp & =P(I=0\wedge E=0\;|\;S^{-}\wedge E_{ps}(I))\\ & \;+\overset{N}{\underset{i=1}{\sum }}P({Ĩ}_{i}=0\wedge E=i\;|\;S^{-}\wedge E_{ps}) \end{array}$$

with

$$\begin{array}{ll} & {Ĩ}_{dp}=I\\ & {Ĩ}_{hy}=I_{hy}\\ & {Ĩ}_{wm}=I(f(J_{1},\ldots ,J_{T-n}))=\left\{\begin{array}{ll} 1\; & \text{if}\;f(J_{1},\ldots ,J_{T-n})>z\\ 0\; & \text{else} \end{array}\right.\\ & {Ĩ}_{ie}=1\vee {Ĩ}_{ie}=0\\ & {Ĩ}_{sb}=\left\{\begin{array}{ll} 0\; & \text{if}\;D^{+}\\ 1\; & \text{if}\;D^{-} \end{array}\right. \end{array}$$

and with $E_{ps}$ representing the IEs that had to be present or absent for the individual being in the stratum (events handled with principal stratum), E∈{0,1,…,N} is the indicator which event type was present (with 0 implies no occurred event) and N is the number of different IE (events handled with the principal stratum strategy where excluded). ${Ĩ}_{i}$ is the used function for the selected strategy if the $i^{th}$ IE occurs.
